# Delayed pain relief in patients with trigeminal neuralgia following microvascular decompression: A single-central retrospective study

**DOI:** 10.3389/fneur.2022.946897

**Published:** 2022-10-19

**Authors:** Zhengyu Zhang, Fang Wang, Feng Yu, Sze Chai Kwok, Jia Yin

**Affiliations:** ^1^Department of Neurosurgery, Shanghai Tenth People's Hospital, Tongji University School of Medicine, Shanghai, China; ^2^Department of Neurosurgery, 960th Hospital of PLA, Jinan, Shandong, China; ^3^Shanghai Key Laboratory of Brain Functional Genomics, Key Laboratory of Brain Functional Genomics Ministry of Education, Shanghai Key Laboratory of Magnetic Resonance, Affiliated Mental Health Center (ECNU), School of Psychology and Cognitive Science, East China Normal University, Shanghai, China; ^4^Division of Natural and Applied Sciences, Duke Kunshan University, Kunshan, China; ^5^Shanghai Changning Mental Health Center, Shanghai, China

**Keywords:** trigeminal neuralgia, microvascular decompression, delayed relief, neurovascular conflict, root entry zone

## Abstract

**Background:**

Compared to hemifacial spasm after microvascular decompression (MVD), delayed relief (DR) rarely occurs in patients with trigeminal neuralgia (TGN).

**Objective:**

To analyze the characteristics of post-MVD DR in TGN patients to provide useful clues for the clinical differential diagnosis of postoperative DR.

**Methods:**

The clinical data of all patients with TGN who underwent MVD in our center from January 1, 2016, to December 31, 2020, were reviewed retrospectively.

**Results:**

In 272 TGN MVD patients, DR occurred in nine patients (3.3%) during the follow-up periods of 1–6 years. During surgery, all nine DR-TGN patients were identified as having neurovascular conflicts (NVCs), involving the offending artery (OA) in eight patients (two OAs in two patients) and both an artery and a vein in the other patient. The compression site was near the root entry zone (REZ) in most DR patients (7/9). Delayed relief was relieved in seven patients within 5 days after surgery and within 30 days in the other two patients. No recurrence or serious complications were observed within the mean 4 (1-6)-year follow-up duration.

**Conclusion:**

Delayed relief rarely occurs in TGN patients after MVD. Neurovascular conflicts located at the REZ and NVC of grade III may be two important factors contributing to DR in TGN patients. Delayed relief may occur when the pain gradually improves after the operation and responds effectively to a small dose of carbamazepine. The recurrence rate of TGN seems even lower in such patients.

## Introduction

The global incidence of trigeminal neuralgia (TGN) is approximately four per 100,000 individuals (1). The incidence of TGN increases with age, and the number of TGN patients has increased with the aging society (2, 3). In TGN patients who fail to respond to or cannot tolerate pharmaceutical treatment, surgery is often needed. As TGN is typically caused by neurovascular conflicts (NVC), microvascular decompression (MVD) is recommended as the most common and preferred surgical modality for TGN in most of the currently available guidelines (4–8). The most obvious advantage of TGN MVD is immediate pain relief after surgery (9, 10), and partial pain relief or pain relief failure was observed in only a small number of patients. In the early days of using the MVD technique, delayed relief (DR) was observed in some patients but has been rare in recent years (11, 12). However, we noticed in our clinical practice that a small number of TGN MVD patients also experienced a DR phenomenon that was similar to that experienced by patients with hemifacial spasm after MVD. Interestingly, this phenomenon often occurred in patients whose MVD was so successfully performed that even the attending surgeon himself felt satisfied because typical NVC was detected in all these patients during surgery. The purpose of this study was to retrospectively analyze a small series of DR patients with TGN who received MVD in our center, and we hope that our findings could provide useful clues for prognosis assessment when neurosurgeons encounter similar cases in their clinical practice.

## Patients and methods

### Patients

Included in this study were patients with TGN who received MVD in our center from January 1, 2016, to December 31, 2020. The diagnosis of TGN was established based on the diagnostic criteria for classic TGN (13.1.1.1) in the International Classification of Headache Disorders 3 (ICHD-3). This study also included some patients who had previously received other surgical treatments, such as percutaneous radiofrequency thermocoagulation, percutaneous balloon compression (PBC), or γ-knife surgery. Preoperative time-of-flight sequence MRI was performed as part of the routine clinical management of TGN in all the patients. Based on the NVC classification by Sindu (13), some patients with NVC Grades I were excluded from our study. For this reason, the operation performed often used multiple methods of neurolysis to treat trigeminal never root (TNR), including neurocombing or aneurysm clip mutilation (14–16), and these cases were excluded from our study because they no belonged to pure MVD surgery. The current study was approved by the Ethics Committee of the authors' hospitals. As all data were anonymous, informed consent of the patients was not applicable.

### Surgical procedures

Microvascular decompression was performed using a standard suboccipital retro-sigmoid approach. After releasing the cerebrospinal fluid (CSF) under the microscope, the cerebellar hemisphere was retracted, and the arachnoid membrane between the petrosal vein and the facial-auditory nerve was opened to expose the cisternal segment of the TNR. The entire length of the TNR (from the pons to the entrance of Meckel's cave) was dissected to identify the NVC. A Teflon sponge was applied between the trigeminal nerve (TN) and OV to decompression. The surgical field was closed routinely.

The surgical outcome was assessed by the Barrow Neurological Institute Pain Intensity Scale (BNI) (17). The initial BNI assessment was performed during the morning ward rounds on the second day post-operatively and then daily until suture removal 5–7 days after surgery and before discharge. The patients were followed up by routine physical examinations on an outpatient basis at 1, 3, 6, and 12 months after surgery and then yearly afterward. Those patients who failed to visit our clinic at the scheduled time points were followed up by telephone interviews.

Delayed relief was judged according to the following A+B criteria. A: Initial complete pain relief was not reported during the first postoperative ward rounds in the next morning; B: Patients with gradual pain relief categorized as BNI I after time had passed during the follow-up period. These cases were considered partially effective or ineffective when pain persisted during the follow-up period, and the patients were not included in the present study.

## Results

**Figure 1:**
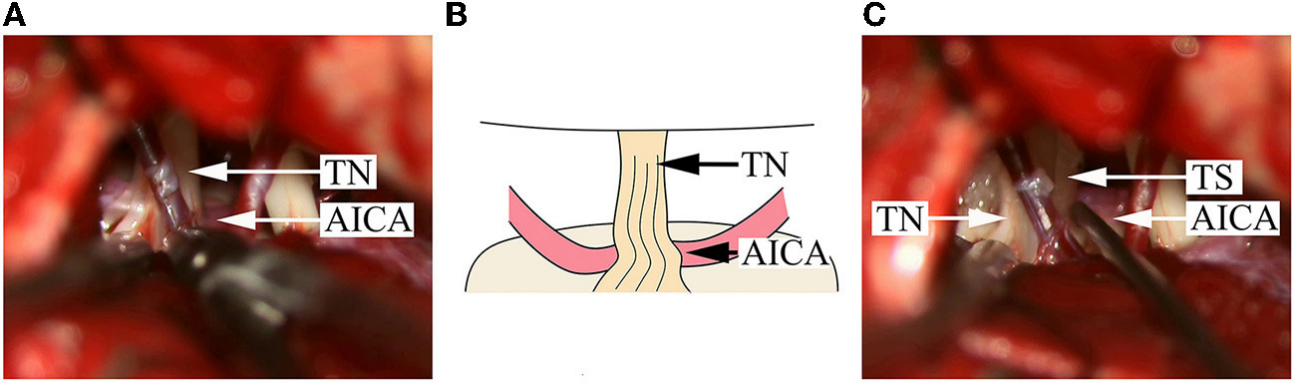
Intraoperative findings in a DR patient with compression from a single offending artery on the root entry/exit zone. **(A)** An AICA is identified; the NVC is located on the ventral side of the TN REZ; the color of the nerve root on the dorsal side becomes bright and is uplifted and distorted upon squeezing. **(B)** A schematical picture of **(A)**. **(C)** After pushing away the AICA, a Teflon sponge is inserted, and the nerve root immediately becomes morphologically normal. AICA, anterior inferior cerebellar artery; TN, trigeminal nerve; TS, Teflon sponge.

**Figure 2:**
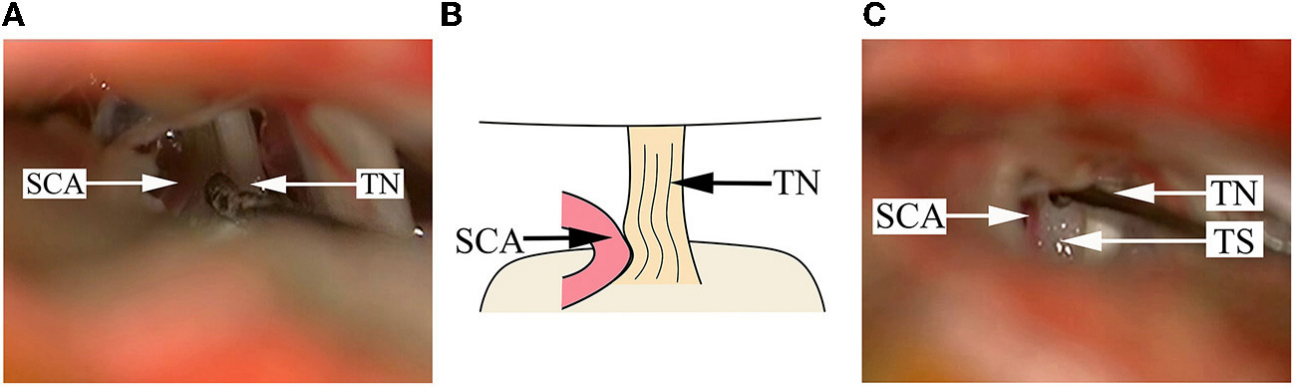
Findings in a DR patient whose cisternal segment of TN is compressed by the offending artery. **(A)** A SCA is impacted into the cisternal segment of TN, and the color of the TN root becomes dark, seriously deformed and depressed. **(B)** A schematical picture of **(A)**. **(C)** After pushing away the SCA, a Teflon sponge is placed between the artery and the TN root. SCA, superior cerebellar artery; TN, trigeminal nerve; TS, Teflon sponge.

**Figure 3:**
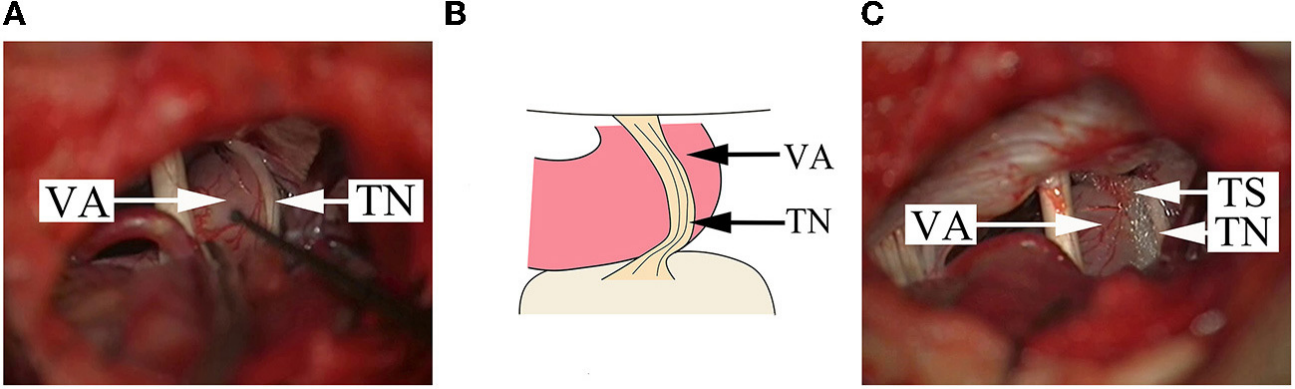
Findings in a DR patient whose offending vessel is a single VA. **(A)** The cisternal segment of TN becomes a flat thin layer because of the serious compression by VA. **(B)** A schematical picture of **(A)**. **(C)** After pushing away the VA from multiple points, a Teflon sponge is placed between the TN and the VA to achieve decompression. VA, vertebral artery; TN, trigeminal nerve; TS, Teflon sponge.

**Figure 4:**
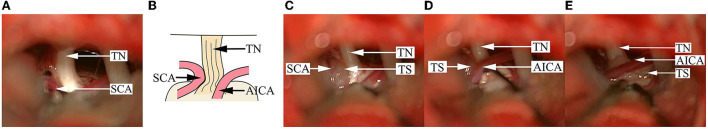
Findings in a DR patient with two offending vessels compressing the TN. **(A)** A SCA is seen compressing the superior aspect of the TN REZ, and the nerve root becomes dark and deformed. **(B)** A schematical picture of **(A)**. **(C)** After pushing away the SCA, a Teflon sponge is inserted between the SCA and the TN. **(D)** Another AICA is seen compressing the dorsal side of the TN REZ, and the surface of the nerve is depressed and changes in color. **(E)** After pushing away the AICA, a Teflon sponge is inserted between the AICA and the TN to achieve decompression. AICA, anterior inferior cerebellar artery; SCA, superior cerebellar artery; TN, trigeminal nerve; TS, Teflon sponge.

**Figure 5:**
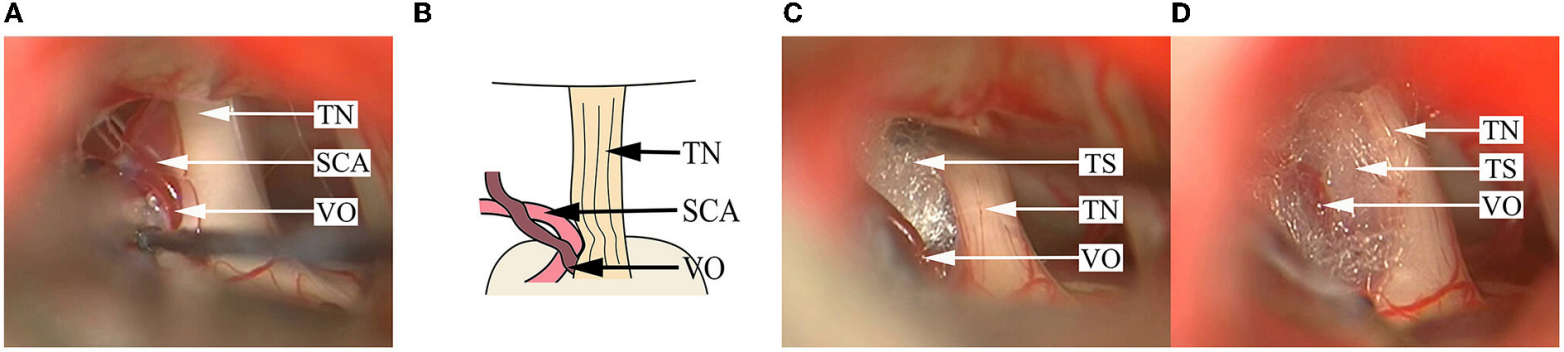
Findings in a DR patient whose OA and vein simultaneously compress the TN REZ. **(A)** A branch of the petrosal vein and an SCA are seen compressing the superior aspect of the TN REZ. **(B)** A schematical picture of **(A)**. **(C)** After pushing away the SCA, the Teflon sponge is inserted between the TN. **(D)** Decompression is implemented by inserting the Teflon sponge between the vein participating in NVC and the TN root. SCA, superior cerebellar artery; TN, trigeminal nerve; TS, Teflon sponge; VO, venous offending.

Of the 272 included patients, pain was relieved immediately after surgery in 252 (92.6%) during the first postoperative ward rounds the next morning; partial pain relief was achieved in 20 patients, and failure was achieved in 0 patients. Of the 20 patients with partial pain relief, 9 patients (3.3%) experienced gradual pain relief and were defined as having DR with the diagnostic criteria above. Analysis of the nine DR patients with complete follow-up data showed that none of these patients had undergone surgical treatment before MVD in our institution. The median age of the nine patients was 67 (49–78) years, and the median disease duration was 3.5 (1–15) years. Of them, five patients presented with symptoms on the left side, and the other four patients presented with symptoms on the right side. The V2 and V3 distributions of the trigeminal nerve were most commonly involved. All nine patients underwent simple MVD without undergoing neurolysis. One offending artery (OA) [one anterior inferior cerebellar artery (AICA) (Figure 1, Supplementary Video S1), four superior cerebellar arteries (SCAs) (Figure 2, Supplementary Video S2), and one vertebral artery (VA) (Figure 3, Supplementary Video S3)] was detected intraoperatively in six cases, two OAs (Figure 4, Supplementary Video S4) in two cases, and an artery and a vein both in the other case (Figure 5, Supplementary Video S5). The NVC was near the root entry zone (REZ) in seven of the nine cases, mostly on the ventral side (6/9). Two of the nine patients had an NVC grade II, and the other seven patients had an NVC grade III. Pain was relieved to varying degrees within 24 h after surgery in eight of the nine patients compared with their pain before the operations. In one patient, the postoperative pain (BNI V) was similar to the preoperative pain (BNI IV). Five patients with BNI >III began using a small-dose (100 mg) of oral carbamazepine BID starting from day 2 after the operation, and the carbamazepine completely relieved the pain in three of the five patients within 5 days after starting the drug. The longest drug administration to achieve BNI I was near 30 days. The demographic characteristics, treatments, and outcomes are listed in Table 1.

**Table 1:** The demographic characteristics, treatments, and outcomes of the patients in DR group.

**Patient**	**Sex**	**Age**	**Side**	**Territory involved**	**Duration (years)**	**Offending vessel**	**NVC finding**		**Postoperatively**			
							**Grade**	**Position**	**Follow-up (years)**	**BNI**	**Medicine (BID)**	**Relief time (days)**
1	F	68	L	I	4	AICA	III	REZ	6	III	100 mg	5
2	M	72	L	II	3.5	SCA+AICA	II	REZ	6	II	No	3
3	F	55	R	III	1	VA	III	Cisternal	5	II	No	4
4	F	58	L	II	15	SCA+AICA	III	REZ	4.5	III	100 mg	14
5	M	49	R	II+III	1.5	SCA	II	REZ	4	IV	100 mg	2
6	F	73	R	II+III	2	SCA+vein	III	REZ	3	II	No	30
7	F	67	L	III	3	SCA	III	Cisternal	2.5	III	100 mg	5
8	M	53	L	I+II+III	4	SCA	III	REZ	2.5	II	No	2
9	F	78	R	II+III	6	SCA	III	REZ	1	III	100 mg	3

All nine DR patients recovered well postoperatively, had the sutures uneventfully removed as scheduled, and were discharged from the hospital within a week after surgery. No intracranial hemorrhage, infection, CSF leakage, or failure of wound healing occurred in any patient. One patient experienced facial numbness, which was cured spontaneously 1 month after surgery. Another patient had a 20-db hearing loss, which was not treated specifically. No recurrence was observed in any of the nine DR patients during the mean 4 (1–6)-year follow-up period.

## Discussion

The immediate pain relief from MVD is very high to such a point that DR is not unanimously recognized in TGN cases (as opposed to HFS patients). Some neurosurgeons do not acknowledge the presence of DR in TGN MVD and additionally, they regard immediate pain relief as the advantage of MVD compared with r-knife and PBC for the treatment of TGN (21–23). Based on the results observed in our center, DR is a matter of fact. In addition, it was also once reported such a phenomenon in the literature.

We used “TGN, MVD, and delayed” as the index terms to conduct a literature review in PubMed and found one study by Deng et al. that discussed DR in detail (24). Their article summarized 105 patients, of whom 20 patients (19%) experienced DR. Statistical analysis revealed no factor that could predict the occurrence of DR, and DR did not affect the long-term effects observed in these patients. They recommended that patients should be monitored for approximately 3 months after MVD and/or PSR and then should be evaluated for the surgical effects; no reoperations should be performed immediately.

The other studies that have mentioned DR include Wang et al. and Shi et al. (9, 25), in which they reported a DR rate of VO cases was higher than the rate of OA cases. Li et al. (12) compared 45 patients with typical presentations and 17 patients with atypical presentations. And indicates that the result of typical TGN is better than that of atypical TGN, but there is no further information about DR. Of the 23 patients reported by Wang et al. (26), 4 patients with VA OV experienced transient partial pain relief (BNI II–III), and 3 (75%, 3/4) patients achieved complete pain relief within 3 months, suggesting that DR may be related to VA.

In this article, we summarized the general data, intraoperative findings, and postoperative outcomes of these nine DR patients and compared them with the other patients from the same period. The details of these data and the results of the literature review are shown in Table 2. However, we were unable to perform a comprehensive analysis due to the limited number of cases.

**Table 2:** General data and intraoperative findings of DR patients and literature review.

		**DR group**	**The others**	**Literatures**
		**(*n* = 9)**	**(*n* = 308)**	(*n* = 1,185) (18)^*^
Age (years), median (range)		67 (49–78)	65 (32–84)	57 (5–8)
Duration (years), median (range)		3.5 (1–15)	4 (0.5–30)	6 (1–44)
Female sex, no. (%)		6 (66.7%)	203 (65.9%)	706 (59.6%)
Affected side, no. (%)				
	Right	4 (44.4%)	159 (51.6%)	724 (61.1%)
	Left	5 (55.6%)	149 (48.4%)	442 (37.3%)
Territory involved, no. (%)				
	I	1 (11.1%)	9 (2.9%)	33 (2.7%)
	II	2 (22.2%)	40 (13.0%)	213 (17.7%)
	III	2 (22.2%)	68 (22.1%)	176 (14.6%)
	I, II	0 (0%)	21 (6.9%)	207 (17.2%)
	II, III	3 (33.3%)	108 (35.1%)	427 (35.5%)
	I, II, III	1 (11.1%)	62 (20.1%)	148 (12.3%)
OV, no. (%)				
	SCA	4 (44.4%)	121 (39.3%)	909 (75.5%)
	AICA	1 (11.1%)	34 (11.0%)	116 (9.6%)
	VA	1 (11.1%)	29 (9.4%)	19 (1.6%)
	SCA+AICA	2 (22.2%)	51 (16.6%)	None
	Others	1 (11.1%)	73 (23.7%)	160 (13.3%)
Grades of NVC, no. (%)				(*n* = 560) (19)
	I	0 (0%)	37 (12.0%)	99 (17.6%)
	II	2 (22.2%)	174 (56.5%)	275 (49.2%)
	III	7 (77.8%)	97 (31.5%)	186 (33.2%)
Position of NVC, no. (%)				(*n* = 8) (20)
	Cisternal	2 (22.2%)	146 (47.4%)	30 (34.9%)
	REZ	8 (77.8%)	162 (52.6%)	56 (56.1%)

* The first literature cited in table, 19 patients who underwent bilateral operation, are counted twice in some tabulations.

As shown in Table 2, there were no significant differences in age, sex, disease duration, territory involved, or side between the DR patients and non-DR patients of the same period and those patients retrieved from the literature review. The main intuitive and obvious characteristics of the DR patients we found are as follows:

1) The grades of OA compression in NVC were relatively high;
2) Most NVCs were near the REZ (7/9);
3) The postoperative BNIs were II–III in most cases (8/9), and oral administration of small-dose carbamazepine for relieving DR;
4) Pain lasted for a relatively short period in most cases (7/9 ≤ 5 days);
5) No recurrence was observed during the follow-up period. Nevertheless, there may be bias in the above results because of the small sample size.

This study has some limitations. First, it is a retrospective study. As the sample size was relatively small, we were unable to perform a statistical analysis, which may affect the reliability of the conclusion. In addition, the follow-up duration was not long enough.

In conclusion, DR may occur in a small number of TGN patients following MVD, possibly due to severe arterial compression (NVC grade III) near the REZ. In cases in which the pain is gradually relieved during the patient's hospital stay and is responsive to oral administration of small-dose carbamazepine, DR should be suspected, and in such patients, the recurrence rate seems even lower.

## Data availability statement

The raw data supporting the conclusions of this article will be made available by the authors, without undue reservation.

## Ethics statement

The studies involving human participants were reviewed and approved by Ethics Committee of Shanghai Tenth People's Hospital. Written informed consent for participation was not required for this study in accordance with the national legislation and the institutional requirements.

## Author contributions

JY conceived and designed the study. ZZ and FY wrote the paper. FW revised the manuscript. SK reviewed and edited the manuscript. All authors read and approved the manuscript.

## Funding

This work was supported, in part, by the National Natural Science Foundation of China (#81671201 to JY and #32071060 to SK) and Tenth People's Hospital clinical research funding (YNCR2A002 to JY).

## Conflict of interest

The authors declare that the research was conducted in the absence of any commercial or financial relationships that could be construed as a potential conflict of interest.

## Publisher's note

All claims expressed in this article are solely those of the authors and do not necessarily represent those of their affiliated organizations, or those of the publisher, the editors and the reviewers. Any product that may be evaluated in this article, or claim that may be made by its manufacturer, is not guaranteed or endorsed by the publisher.

## Supplementary material

Supplementary Video S1 An AICA is seen on the ventral side of the TN REZ; after pushing the TN root outward, the lateral side of the TN root turns bright and becomes uplifted and deformed upon pushing and squeezing. After separating the AICA from the TN root with the nerve stripper and pushing the AICA to the medial side, a Teflon sponge is placed between the artery and the TN root. The operation ends when the TN root becomes morphologically normal.

Supplementary Video S2 An SCA is seen in the superior aspect of the cisternal segment of TN, which arcuately compresses and compacts into the TN sensory root, and the compressed part becomes dark in color, seriously deformed and depressed. After pushing away the offending artery, a Teflon sponge is placed between the artery and the nerve root to achieve decompression. The operation ends when the TN root becomes morphologically normal.

Supplementary Video S3 A large and thick VA is seen in the inferior aspect of the TN, which is compressing the TN, causing serious deformation of the TN, and the TN becomes a flat thin layer. The nerve trippers are used to support the VA from multiple points to see whether it could beneficially reduce the compression force on the TN. Teflon sponges are inserted between the offending artery and the brain stem and at multiple points between the petrosal bone surfaces. When the compression force of VA on TN becomes significantly smaller, a Teflon sponge is inserted to effect decompression. The operation ends when satisfactory decompression is achieved.

Supplementary Video S4 An SCA is seen compressing the superior aspect of the TN REZ, and the TN root becomes dark and deformed. After pushing away the SCA, a Teflon sponge is placed between the SCA and the TN. Simultaneously, another AICA is seen compressing the dorsal side of the TN REZ, and the nerve surface is depressed and changes in color. After pushing away the AICA, a Teflon sponge is placed between, and the operation ends as scheduled.

Supplementary Video S5 An SCA and a branch of the petrosal vein are seen compressing the superior aspect of the TN REZ. The SCA is mainly responsible for the compression. A Teflon sponge is inserted between the SCA and the TN root after pushing away and between the vein participating in NVC with the nerve root. The operation ends when satisfactory decompression is achieved, and the morphology of the TN root becomes normal.
